# Identifying social determinants of health in populations exposed to structural inequities: a qualitative study of the COVID-19 pandemic experiences of Black and Latinx people living with HIV and cardiovascular risks

**DOI:** 10.3389/fpubh.2024.1336184

**Published:** 2024-05-30

**Authors:** Tamra Burns Loeb, Graciela I. Albarran, Ethan Lee, Jenna Alarcon McEwan, Karen E. Dyer, Michele Cooley-Strickland, Enricka Norwood-Scott, Kate Ramm, David Kesblyan, Aleeja Barnes, Derek Novacek, Dorothy Chin

**Affiliations:** ^1^David Geffen School of Medicine, University of California, Los Angeles, Los Angeles, CA, United States; ^2^Research Volunteer, University of California, Los Angeles, Los Angeles, CA, United States; ^3^Independent Researcher, Los Angeles, CA, United States; ^4^Loyola University Chicago Stritch School of Medicine, Maywood, IL, United States; ^5^Independent Volunteer, Los Angeles, CA, United States; ^6^Desert Pacific Mental Illness Research, Education, and Clinical Center, Veterans Affairs Greater Los Angeles Healthcare System, Los Angeles, CA, United States

**Keywords:** SDOH, inequities, COVID-19, public health information, vaccine uptake, lived experience

## Abstract

**Introduction:**

Black and Latinx communities experience inequities in the social determinants of health (SDOH) and high rates of chronic illnesses [e.g., cardiovascular disease (CVD), HIV]. The COVID-19 pandemic amplified these long-standing SDOH disparities. However, scant attention has been paid to the pandemic-related experiences of populations exposed to structural inequities.

**Methods:**

Using a semi-structured interview guide, 60 in-depth telephone interviews were conducted with Black and Latinx people living with HIV (PLWH) and CVD risks to assess: (1) perceived personal and community risk for COVID-19; (2) knowledge of and access to COVID-19 public health information; (3) barriers to COVID-19 public health recommendations and vaccine uptake; and (4) perceptions of HIV, CVD, and COVID-19. Interviews were professionally transcribed into either English or Spanish. Spanish transcripts were translated into English. Rapid qualitative analysis was used to summarize each transcript into a structured templaicte corresponding to interview guide domains. Summaries were combined into matrices for identification and comparison of themes across domains.

**Results:**

Participants reported risks for COVID-19 due to being immunocompromised and SDOH, including transportation, exposure to risks conferred by others, living in under-resourced neighborhoods, and housing insecurity. Participants engaged in protective countermeasures by adhering to public health mandates. Relationships with providers, participating in community support groups, and digital inclusion and literacy were salient with respect to dissemination of COVID-19 information and vaccine uptake. Experiences with managing a chronic illness facilitated vaccine acceptance. Participants described language barriers, experiences of discrimination, and a historical lack of trust in medical systems and vaccines.

**Discussion:**

This study provides a real-time narrative from PLWH and CVD risks who were vulnerable during the height of the COVID-19 pandemic. Implications include the need for continuity with providers and established community networks, increasing internet access and digital health literacy, and addressing historical trauma incurred in medical settings. It is critical to understand the impact of traditional SDOH on those living with chronic illness as well as other social determinants that shed light on access to public health information, adherence to public health recommendations, and vaccine uptake among populations exposed to structural inequities.

## Background

Since the founding of the United States, individual action, policy choices, and/or nationally organized efforts have marginalized certain groups (e.g., Indigenous people of North America, Black/African Americans, Hispanic Americans/Latinx), creating inequities in structure and access to resources, wealth, and power (1). As a result, Native American, Black, and Latinx communities have long experienced inequities in the social determinants of health (SDOH), the factors under which people are “born, grow, live, work, and age” (2, 3). Black American and Latinx people also experience the highest rates of chronic illnesses (e.g., cardiovascular disease, HIV) among all racial and ethnic groups in the United States (4–6).

The COVID-19 pandemic and subsequent public health response amplified these long-standing SDOH inequities, particularly among those at highest risk for COVID-19 (7–10), further heightening risks for morbidity and mortality (9). Despite research that documents these complex relationships [i.e., (7, 10, 11)], scant attention has been paid to understanding the pandemic-related experiences of structurally vulnerable populations and identifying salient social determinants that impact their wellbeing (12, 13). Inattention to the lived experiences of vulnerable subpopulations – already disproportionately impacted by the intersection of SDOH, chronic illness, and heightened COVID-19 risks – severely restricts the ability of healthcare systems, programs, and policies to address both short- and long-term needs as well as prepare for future public health threats (14, 15).

*Need for research to identify salient SDOH in vulnerable populations during the COVID-19 pandemic*.

The World Health Organization and Healthy People 2030 have identified five major domains of SDOH: Economic stability; education access and quality; health care access and quality; neighborhood and built environment; and social and community context (16, 17). The pandemic spurred a new body of research examining how SDOH contributed to – and oftentimes exacerbated – emerging COVID-19 disparities (11). The differential, complex and reciprocal relationships among various SDOH and COVID-19 risk factors and outcomes likely vary across subpopulations. To achieve health equity, we must work to identify social determinants that are particularly salient in specific subpopulations and remain open to the possibility that they may fall outside of the five standard domains.

Consistent with this approach, researchers have begun to expand beyond traditional conceptualizations to include separate, overarching influences on SDOH such as structural inequities and racism (10, 18). Others have identified “super determinants” of health, factors that influence health outcomes and also impact traditional SDOH (e.g., digital inclusion impacts employment, education, and healthcare access) (19). For vulnerable populations living with chronic illness, inequities stemming from structural racism and digital disparities can limit knowledge of and access to public health messages. This is true even for information that is intentionally designed for them because reduced technology access and literacy is not adequately considered, such as when information is posted on government websites (20). The extent to which high-risk subpopulations were both aware that public health messages tailored to them existed and were able to access them during the pandemic is currently unknown. Identifying subpopulation-specific social determinants that affect health outcomes is a necessary strategy to tailor future disease prevention and mitigation efforts.

*Understanding COVID-19 lived experience is central to identifying salient SDOH in populations with structural inequities*.

Population-level community-centered assessment has highlighted the importance of tailoring public health communication strategies to reach specific racial, ethnic, and cultural groups as well as to address SDOH barriers. During the COVID-19 pandemic, large scale research efforts [e.g., the National Institutes of Health (NIH) Community Engagement Alliance (CEAL)] collected qualitative data from community-academic teams and survey data from academic investigators and community-based organization (CBO) partners (12, 21). While CBO partners or community experts (22) serve as important informants and dissemination partners due to their active and ongoing engagement with vulnerable populations, understanding the *lived experiences of populations exposed to structural inequities during public health crises* are a critical component of public health mitigation efforts that must also be prioritized.

The limited resources and barriers to care experienced by populations exposed to structural inequities further impedes researchers’ ability to reach and engage these communities, resulting in fewer studies that meaningfully assess their lived experiences. Qualitative research is an ideal method for documenting and describing the in-depth perceptions, lived experiences, and intersecting inequities experienced by hard-to-reach populations that may be poorly understood or have received scant research attention (23, 24). The historical lack of attention paid to subpopulations exposed to structural inequities limits our ability to understand the impact of SDOH on health behavior risk and protective factors. In a recent review of studies focused on SDOH and COVID-19, none utilized qualitative research methodologies (7), highlighting the need for research that describes the perceptions and experiences of populations disproportionately affected by SDOH and COVID-19. Understanding the salience of these factors on structurally vulnerable individuals’ ability to navigate challenges related to reducing COVID-19 risks is a necessary step to tailor policies, programs, and public health messages to mitigate the spread of COVID-19 and future public health threats.

To address this gap, we used qualitative methods to assess the COVID-19-related perceptions and experiences of Black and Latinx PLWH and cardiovascular disease (CVD) risks in Los Angeles County, California during the COVID-19 pandemic. These included perceptions of risk for COVID-19 and sources of and access to COVID-19 information. Also assessed were a variety of factors that could influence adherence to COVID-19 public health recommendations (e.g., knowledge of and access to reliable COVID-19 information; digital inclusion; past experiences with vaccines; access to transportation; and concerns specific to HIV and CVD as comorbidities).

## Methods

### Study design and sample

Using a semi-structured interview guide informed by the study’s aims, in-depth telephone interviews were conducted with Black and Latinx PLWH and CVD risks to assess 4 predetermined interview guide domains, including (1) perceptions of risk; (2) knowledge of and access to reliable sources of COVID-19 public health information; (3) barriers to COVID-19 public health recommendations and vaccine uptake; and (4) personal experiences related to HIV, CVD, and COVID-19. Participants responded to several questions within each domain (see Table 1 for examples of interview questions).

**Table 1:** Interview guide domains and examples of questions.

Domain	Examples of interview questions
Perceptions of risks	I’d like to know your thoughts about risks for COVID-19. Do you think you are at risk for COVID-19? Why or why not? Do you think your community is at risk for COVID-19? Why or why not? Are you fully vaccinated against COVID-19?
Knowledge of and access to reliable sources of COVID-19 public health information	I’d like to ask you where you get your information about COVID-19 prevention, testing, and vaccines from. What is your main source of COVID-19 information? (some people get information from their doctor, the internet, television, community leaders, Church, friends, family, social media, etc.) Do you know if there is COVID-19 information designed specifically for people living with HIV?
Barriers to COVID-19 public health recommendations and vaccine uptake	Has anything made it difficult for you to follow COVID-19 public health mandates? For example, keeping 6 feet distance from others, wearing masks, self-isolating if experiencing symptoms, etc. If so, what? Would anything make it difficult for you to get a COVID-19 test, if you wanted one? If so, what? Would anything make it difficult to get a COVID-19 vaccine, if you wanted one? If so, what? Have you experienced difficulty getting COVID-19 information in your preferred language? Can you tell me about that? How well are you able to use the internet to get COVID-19 information and services? Do you have access to the internet? If no: How does this affect your ability to get COVID-19 information or services? If yes: Do you use the internet to get COVID-19 information or services? Do you have access to a computer or tablet? What are your thoughts and feelings about vaccines in general? Can you tell me about some of your past experiences with vaccines? Have you had any experience of being discriminated against during the pandemic (for example, wearing a mask or face covering in public)? If so, can you tell me about it? Did that experience affect your ability to get COVID-19 information or services? If so, how?
HIV and COVID-19	Do you feel like you may be approaching COVID-19 differently because of something you learned about taking care of yourself with HIV? Has COVID-19 affected your HIV care? If so, how? Does your HIV status affect the way you think and feel about COVID-19 vaccines? Why or why not? If you have already received the COVID-19 vaccine, who recommended that you get the vaccine?

Participants were all enrolled in an ongoing National Heart, Lung, Blood Institute-funded parent trial entitled, “*Enhancing patient and organizational readiness for cardiovascular risk reduction among Black and Latinx patients living with HIV*” (25). The specific aims for the current study are distinct from those of the parent study (i.e., to increase patient and organizational readiness to address CVD risk reduction). Parent trial participants were recruited from HIV clinics throughout Los Angeles County, California, and were between 18 and 75 years of age, HIV-positive, had no immediate plans to move outside of Los Angeles, spoke English or Spanish, and endorsed a history of trauma/adversity and cardiovascular risk.

### Recruitment procedures

Any participant who enrolled in the parent trial between September 13, 2022, and March 28, 2023, was eligible for participation in the current study and offered the opportunity to engage in an interview. Recruitment continued until the target sample of 60 (40 English-speaking and 20 Spanish-speaking) participants was reached. Project staff contacted potential participants by telephone to introduce the study, assess interest, and schedule interviews at a day/time convenient to participants. Participants received a $30.00 gift card incentive for participation in the telephone interview. The study was approved by the South General UCLA Institutional Review Board, IRB #21-000762.

### Data collection

A total of 60 participants completed interviews in their language of choice between September 2022 and March 2023. Forty English- and 20 Spanish-speaking telephone interviews were conducted. Participants were between the ages of 18–75. Informed consent was obtained verbally following IRB-approved procedures. Interviews were audio-recorded with participants’ permission, and typically lasted between 30 and 45 min.

### Data analysis

The research team used Rapid Qualitative Analysis (RQA) as their analytic approach, which is a type of manifest content analysis developed for and utilized in health equity research to quickly identify and address the needs of marginalized communities (26–28). While more traditional and well-known qualitative approaches (such as Grounded Theory) are often highly resource-intensive and lengthy processes, RQA on the other hand is an “action-oriented approach to qualitative data analysis that may be used when findings are needed to quickly inform practice” (St. George 2023:1). RQA is particularly well-suited to the present study, which seeks to assess the lived experiences and needs of a vulnerable community in the midst of the COVID-19 public health emergency. The research team followed an established, well-delineated RQA approach developed by Hamilton and colleagues, which has been employed in a range of health equity projects and health services research studies (26, 27, 29, 30).

All interviews were digitally recorded and professionally transcribed into either English or Spanish. Spanish transcripts were then translated into English by a culturally bilingual staff member. After transcripts were reviewed in detail by all research team members, the next step involved summarizing each transcript into a structured “interview summary template.” Condensing data in this fashion allows qualitative researchers to assess the depth and breadth of the available data under each interview domain, to identify preliminary themes or areas needing further exploration, and/or to guide subsequent data collection and analysis strategies (30). The study’s PI and co-Investigators drafted an initial summary template that generally corresponded to the main interview guide domains depicted in Table 1 (i.e., perceptions of risk; knowledge of and access to reliable sources of COVID-19 public health information; barriers to COVID-19 public health recommendations and vaccine uptake; and HIV, CVD, and COVID-19). This initial draft was refined through an iterative process, whereby each member of the analysis team independently summarized the same “test transcript,” then met as a group to discuss areas of agreement/divergence on how well the template captured the intended domains. The draft was revised accordingly, and the process was repeated with two additional transcripts until the group reached consensus on a final template version. The remaining transcripts were then divided up amongst team members to summarize independently using the final summary template.

After all interview summaries were completed, the analysis team created a set of matrices organized by topic to more efficiently identify trends across respondents (28). After grouping related interview domains of interest into separate matrices, cells were populated with domain-specific data from each individual interview summary (i.e., “respondent x domain”) (29). Team members performed matrix analysis to identify key themes and takeaway points by comparing responses across interviews and identifying illustrative participant quotes for each finding (28, 29).

## Results

Results are presented corresponding to each of the four interview guide domains. Each domain included questions related to that topic. These included: (1) perceptions of risk (questions related to personal and community risk for COVID-19, and vaccine status); (2) knowledge of and access to reliable sources of COVID-19 public health information (questions about sources of COVID-19 information and knowledge of COVID-19 information for PLWH); (3) barriers to COVID-19 public health recommendations and vaccine uptake (questions about barriers experienced, vaccine-related perceptions and experiences, and experiences of discrimination and their potential impact on HIV care); and (4) HIV, CVD, and COVID-19 (questions concerning the influence of HIV on participants’ approaches to COVID-19, the impact of COVID-19 on HIV care, perceptions of the COVID-19 vaccine related to HIV and CVD, and sources of COVID-19 vaccine recommendations).

### Perceptions of risk

#### Personal risk for COVID-19

Participants were asked whether they thought they were at risk for COVID-19 and to explain their reasoning. Over three-quarters of participants stated they felt personally at-risk for contracting COVID-19, whereas 14 participants reported no personal risk. Despite almost all of the participants indicating that they were fully vaccinated against COVID-19, over two-thirds perceived personal risk for COVID-19. Over 20% of the sample reported being vaccinated with no remaining personal risk. Five participants described being unvaccinated and having personal risk for COVID-19; 4 of the 5 were English-speaking. One participant reported being unvaccinated but without personal risk.

##### Themes among participants perceiving personal risk for COVID-19

Among those participants who voiced having personal risk for COVID-19, four main themes were described. These included having a compromised health status, the ongoing nature of the pandemic, continued risk despite personal adherence to public health measures, and risks due to exposure to others. Many participants noted that their health status conferred risk (i.e., being immunocompromised, living with HIV and/or other comorbidities such as asthma, diabetes, or cardiovascular disease). For example, one participant noted that, “*I do believe that I’m at risk for it (COVID-19) because of my health issue, my underlying health issue with having full-blown AIDS at this time.*” Second, participants identified personal risks stemming from the ongoing nature of the pandemic (i.e., airborne transmission, “*always there*,” the existence of variants). One participant noted that the virus is “*still out there, mutating*.” Third, many participants discussed feeling like they remained at risk despite personally adhering to public health recommendations. According to one participant, “*Yes, I believe everybody’s still at risk for COVID-19. Even though I’ve been fully vaccinated, you know, I still use precaution because I’ve known someone that has been fully vaccinated but still got COVID*.” The last major reason was the risk presented by exposure to others. For instance, others may not be vaccinated, may not believe in the existence of COVID-19, do not adhere to public health measures, or have jobs that confer risk such as essential workers. According to one participant, “*I do think I’m at risk of getting COVID and the reason, not because of me, but because of others who are not conscientious.*”

##### Themes among participants with no perceived personal risk for COVID-19

Two primary themes were noted among those that described themselves as without risk, including being fully vaccinated and following public health measures (i.e., isolating, wearing a mask). For example, one participant noted, “*…I protect myself because I use the bus a lot, I use my mask even when others do not, I use it. I feel more protected with it*.”; “*I follow all the protocols, well to a T*”; “*The person I live with wears a mask all the time...*” Several participants described being exposed to COVID-19 and not contracting it. One participant noted that they felt they had immunity due a prior COVID-19 infection.

#### Community risk for COVID-19

Participants were asked whether they thought their community was at risk for COVID-19. The definition of “community” was not prespecified for participants, who inductively identified the “community” to which they felt a sense of belonging. Participants identified with several communities, including older age, racial/ethnic minority affiliation, neighborhood in which they lived, and sexual orientation. Most participants indicated that their community was indeed at risk for COVID-19, while fewer indicated no risk or were unsure. The proportion of English-speaking participants who did not endorse community risk was double the proportion found among Spanish-speaking participants.

##### Themes among participants perceiving community risk for COVID-19

Five primary themes were reported by participants who perceived community risk. These included community members failing to follow public health mandates, the socio-geographic characteristics of the community, the ongoing nature of the pandemic, a lack of reliable public health information available in the community, and distrust in the medical system and vaccines among community members. The most prominent theme involved some community members failing to take precautions (i.e., not following published recommendations or relaxing adherence to public health mandates). One participant explained, *“Because a lot of people do not use their masks, they do not take care of themselves, and they do not get the vaccines. A lot of people still have not been vaccinated.”* Others noted that some community members no longer take the pandemic seriously; over time they “*have let their guard down and aren’t taking precautions,”* display a *“false sense of security,” or* “*do not wear masks anymore or social distance [and] act like COVID is gone.”*

A second theme voiced by participants stemmed from their community’s socio-geographical characteristics, including the state, neighborhood, or housing type. One participant perceived that [my community is at risk] “*because in California, moving around is obligatory and that is where the risk is: when you have to move around*.” Participants also described their communities as under-resourced, such as, “*Because [my community is] poor, it’s poorer than other neighborhoods,”* or as vulnerable (i.e., the gay community living with HIV is immunocompromised). Some described needing to work outside the community thereby increasing their interactions with others. Others described being exposed to risky individuals within their community (i.e., high resident turnover due to living in a shelter). One participant described the contrast between the area where they lived and other, more affluent areas, noting, *“I live in an area where it is very dangerous to be exposed; I live on Skid Row...Okay there are a lot of people who use drugs, they do not take care of themselves, that’s why I’m saying that I always use a mask even though, in that area… because in other areas like where you work, they do not really use that anymore, no? I guess because they are well protected…”* Another noted, *“Well, since I’m in transitional housing, we get a lot of different people. So they have more risk factors because we have intakes almost every day where new people are coming and going. So with that, I’m more at risk.*”

Similar to perceptions about personal risk, another theme reported by some participants centered on the ongoing nature of the pandemic. They noted that COVID-19 is “*still ongoing*,” and [there are] “*new* var*iants*.” Others stated that “*everybody is at risk, breathing the same air*,” and “*COVID is a virus that is in the air everywhere and anyone can have it and they do not tell you.*”

A fourth theme associated with community risk was a lack of reliable public health information, problems with unclear public health messaging, and ignorance. One participant lamented, “*most of them will not have the information to know what to do correctly to help prevent themselves from getting [COVID-19] or being at risk*.” Another explained that “*a lot of people do not get vaccinated, and a lot of people do not believe or aren’t careful or they are ignorant and take risks*.”

The fifth theme voiced by participants involved distrust of the medical system and vaccines in general. One participant voiced that people should not be forced to take a vaccine, as the government does not own other peoples’ bodies. Another described having “*an inkling something was up*” with the COVID vaccine; “*if they are paying the public to get it, which I’ve never heard of or seen before with any other vaccine, it made it kind of fishy and made me not want to trust it more*.” Another participant noted, “*[in] my culture, a lot of them do not want to get the [COVID] vaccine because they are skeptical about whether COVID is real, or they just do not want to keep putting all this stuff into their system. And a lot of times they keep bringing up the study, the Tuskegee study—I think that was the name of it, right? Gave all the Black men an illness and did not tell them and stuff like that, so you know, that sticks with a lot of people in my culture*.” Finally, some participants linked risks in the community to others’ personal behaviors (i.e., sexual behaviors, drug use) and mental health issues (i.e., boredom, isolation, depression).

### Knowledge of and access to reliable sources of COVID-19 public health information

#### Main sources of COVID-19 information

Participants were asked about their main source(s) of COVID-19 information. They provided five general sources of COVID-19 information, including primary care physicians and healthcare providers, the internet, the news, community groups, support groups, or community events focused on COVID-19, and word of mouth. Over two-thirds indicated their main source of COVID-19 information was their primary care physician or other healthcare provider. One participant stated, “*Well, nowadays, wherever you look there is information about COVID. On the radio, the television, your cellphone, everything is full of information… [I get my information] mostly from my primary doctor.*”

One-half of participants used the internet to access COVID-19 public health information, though it was more common among English-speaking participants than Spanish-speaking. Participants sought information from a variety of websites, including the Los Angeles County Health Department, the U.S. Centers for Disease Control and Prevention (CDC), and other local or national government websites (i.e., “.gov”). As one noted, “*I go online and I get alerts from the LA County. And then, …The CDC website and just going to events in the community*.”

More than half of participants reported relying on the news for public health information (primarily TV or radio news, due to limited internet access). More English-speaking than Spanish-speaking participants used the news as one of their main sources of COVID-19 information. One stated, “*mostly [the] media, meaning whatever the latest reports are from CNN. I like to watch CNN… And the news, the regular news channel for the local stuff, if there’s something we should be alerted about or whatever, I’ll look and listen to that*.” Some described needing to be cautious receiving public health information through the news: “*the TV, they sensationalize, so a lot of time they are going to exaggerate*.”

Another one-third of participants received information from community groups, support groups, or community events for COVID-19. One Spanish-speaking participant noted, “*I go to a support group for people with HIV, and sometimes there are doctors or other community members who give us information about COVID-19 and protection.*”

Approximately one-third of participants received COVID-19 information via word of mouth: “*just talking to other people*.” Twelve participants used social media – such as Facebook/Meta – for this purpose, more commonly among English-speaking than Spanish-speaking participants. However, some participants expressed distrust of these sources; as one noted: “*I do not trust secondhand conversations, like social media, because there’s too much false information out there.*” Six participants relied on their place of worship for COVID-19 information, particularly English-speaking versus Spanish-speaking participants. Several participants noted that they relied on multiple sources of information, “*some internet. Like I said, news, media, all over… friends; I get information*.”

#### Knowledge of COVID-19 information for PLWH

Participants were asked about their knowledge of COVID-19 information specifically for people living with HIV (PLWH). More than one-third responded affirmatively, with proportionately more Spanish-speaking than English-speaking participants aware of such information. Participants provided three main responses, including receiving targeted information from HIV-focused groups, lacking information tailored to PLWH, or having to specifically inquire about HIV-focused COVID-19 information. Many participants knew about or belonged to an HIV community group and/or support group that distributed these resources. For example, “*yes, they have literature everywhere, all the clinics—I go to a lot of LGBT community centers, to their programs. And they have tons of literature always out there on the table in the lobby, passing out flyers or people out soliciting [to] get checked or tested or get shots or whatever, yes. They do have it. And they have it designed specifically for Blacks, but not as much, I might say. Not as much for Blacks. They just have it in general*.” Some participants felt that the information available is for the general public, not tailored to the needs of PLWH: “*Well, more than anything it’s directed at the population in general, but I have not seen specific announcements for people who are positive*.” Others stated that PLWH-specific information exists, but one has to go find it or ask for it.

### Barriers to COVID-19 public health recommendations and vaccine uptake

#### Themes related to barriers to following public health mandates

Participants were asked if they experienced any difficulties following COVID-19 mandates. A little over half of the participants described having no problems. Participants shared how they protected themselves by wearing masks, staying at home when possible and through social distancing. Participants who reported experiencing barriers to adhering to public health mandates primarily described challenges due to transportation issues and their living situation. Nearly half of the participants described following the mandates as challenging; most ascribed the difficulties to their mode of transportation. For example, “*buses are a risk because they are too full, and you cannot keep socially distant.*” Another common theme was related to participants’ living situations. Some participants who were incarcerated during the pandemic described unsanitary conditions (e.g., people sleeping on the floor). Another participant noted, “*I was homeless, I do not have anything, so I have to go out and interact just to survive*.”

#### Themes related to barriers to COVID-19 testing and vaccines

Participants were also asked to describe any barriers experienced obtaining COVID-19 testing or vaccines. The majority stated that they had no problems receiving a COVID-19 test, if they wanted one. Those who indicated difficulty obtaining a test were all English-speaking. Barriers included a lack of testing sites in the community, transportation issues, and concern about vaccines given one’s health status. One participant stated that “*there’s far fewer sites to test now in my community than there were even three or four months ago. I’d have to drive around, look on the internet, go long distances*.” Another stated, “*the difficulty is just transportation and knowing the spot to go to.*” The majority of participants were able to obtain COVID-19 vaccines, although a few reported barriers: “*the challenge would be going there because sometimes we do not have transportation*.” In referring to instructions for who could be vaccinated when the vaccines were first rolled out, a participant stated, “*I do not know what category I was and unsure what vaccine does to me with my condition*.”

#### Themes related to language barriers

Participants were asked if they experienced any difficulty accessing COVID-19 information in their preferred language. A high percentage of participants, with proportionately more English-speaking than Spanish-speaking, expressed no difficulty. For example, “*No, not me, there’s no problem honestly because, for example, Spanish, everyone here speaks Spanish. So, the authorities are aware that there are a lot of Hispanic people, the authorities are aware that they need to focus on them, give them information, so information in Spanish is abundant*.” Barriers included a lack of COVID-19 information in Spanish at the onset of the pandemic as well as COVID-19 information tailored to specific racial/ethnic populations. According to one participant, “*I have difficulties with the information because it does not specify for African Americans—so we understand how it affects African Americans. Instead of using it as an umbrella and say, okay, let us have different fliers, different format, not discrimination, but it’s just a different format targeted for those that need African American or those of color*.” Of the participants who encountered difficulties obtaining information in their preferred language, half were Spanish speakers. Another stated, “*I was educated in Spanish, and the person was explaining it to me in Spanish, but there wasn’t a lot of information in Spanish at that time, everything was mainly in English. Now yes, I think there’s more or less COVID information in Spanish… But yes, at first everything was, it was very difficult*.”

#### Themes related to discrimination

Participants were asked if they had been discriminated against during the pandemic and if so, whether it affected their ability to obtain COVID-19 information and services. Over one-third of participants reported experiencing discrimination during the pandemic. One participant noted, “*I’m always discriminated against, mask or no mask, COVID or no COVID, okay?*” Most participants reported issues related to wearing masks. According to one participant, “*Yeah, earlier in the pandemic when I would travel to certain communities, people would give me weird looks because I had on a mask and no one else in the area did. I’m also African American and these people I’m referring to were not. They were Caucasian. So, it seemed like there was double/a little... hostility.*” However, most participants thought that it did not affect their ability to get COVID-19 information or services.

#### Themes related to digital inclusion

Participants were asked if they had access to a computer or tablet, and how well they could use them to get COVID-19 information and services. Barriers included lack of access to digital devices and low digital literacy. Over three-quarters of the participants had access to a computer or tablet, whereas the remaining participants did not. Even those with electronic devices described difficulties using them, “*I’m very* – *what’s the word—illiterate, is that the word they use? I do not know what word they use because I cannot do nothing on the computer. For me it’s very difficult to use a computer. If I try to get something done, believe me, it’s going to take me 24 h to get it done. I’m very bad with computers*.”

#### Vaccine-related perceptions and experiences

##### Themes related to vaccines

Participants were asked to share their perceptions about vaccines in general, as well as past experiences with vaccines. Many participants also used this as an opportunity to expand on their attitudes about and experiences with the COVID-19 vaccine in particular. Beyond expressing a general support of, or opposition to, vaccines, participants’ responses coalesced around three main vaccine-related themes: misinformation and confusion, trust, and physical and emotional trauma.

First, regardless of positive or negative perceptions about vaccines in general, participants described being inundated with misinformation and expressed confusion regarding the COVID-19 vaccine. One participant stated, “*I do not know what the vaccine can do to my body or my meds. I do not know if I take COVID seriously, if I did, would be vaccinated. If a vaccination is going to do something that’s going to mess with that then I do not want to be vaccinated*.” Many participants mentioned that they had received the COVID-19 vaccine despite this confusion because they understood that it would protect them against severe illness or death. One participant noted, “*for example, like the COVID [vaccine], it was necessary because so many people died, and yes, a lot of friends of mine and family died, so I decided to get it, even though I did not know what it was or what it was for*.” Another participant stated “*it makes me more accepting of medication for sure and all of those necessary things prescribed by my doctor. I follow my doctor’s orders*.”

A second major theme regarding the information and dissemination of vaccines was trust. Although participants were not explicitly asked about whether or not they trusted their doctors and public health messaging about vaccines and their efficacy, many discussed these topics. Among those who did, the majority of participants expressed a lack of trust, while a smaller minority described a general feeling of trust. Many participants who described a lack of trust had still received vaccinations; some described feeling like they had no other option because they were afraid of dying. Those who held positive views of vaccines generally expressed feelings of trust in their doctors, medical and public health institutions, and the government. One participant noted, “*I do trust the CDC and their information*,” and another stated, “*The vaccines in general are good. Thanks to them you feel protected, at least I feel protected, I’m fine. And if I did not have, if my defenses were low and with this illness, because I also have diabetes, I’m just missing the combo to make it three. I have diabetes, I have HIV, and if I did not worry about my health… I do see a lot and I am very interested in the vaccines to stay protected*.” While over half of Spanish-speaking participants expressed a generally positive view of vaccines, a much smaller proportion of English-speaking participants held the same views.

In contrast, those who did not hold positive views of vaccines tended to distrust information provided by medical and public health institutions, and the government. For example, “*Sometimes it’s scary because you do not really know if the government, to control us more… because I remember that we all had the thought that they wanted to insert that GPS or something like it into us, … I have a lot of friends who never got the COVID vaccine. Because of that, because they thought the government and all of that, we did not know if it had the GPS*.”; “*If you get it great, and if not, no. And then I started to realize that people were dying. So, I got it. Because I did not want to*.” Both Spanish-speakers and English-speakers who held this view were in the minority. Many relied on information supplied by friends, family, or their community. Participants acknowledged conspiracy theories and historical discrimination against patients of color by medical institutions as reasons for their beliefs.

Third, some participants identified past experiences of physical and emotional trauma as influencing vaccine acceptance. Participants noted that they had experienced physical symptoms after receiving other vaccines, including drowsiness, soreness or swelling in their arms, runny nose, fever, chills, and body aches, and irritability. Participants recounted almost dying after taking the pneumonia vaccine, getting the flu after the flu vaccine, and feeling sick or weak for 1 to 2 weeks after receiving a vaccine. One participant identified a physical trauma that occurred after receiving a prior vaccine, which deterred them from being vaccinated again for almost 30 years: “*I went to get the flu shot and there was a brand-new nurse there. She pricked me so deep that the pain lasted about four months… It was a terrible pain and I had to use my other arm to lift that arm. So, after that vaccine, I just could not trust people, or that another nurse would do it incorrectly or prick me where she wasn’t supposed to and damage my muscle and have this happen all over again. So, after that, I just got used to not accepting vaccines*.”

For other participants, the absence of physical side effects contributed to their positive views of vaccines: “*I’ve not had any negative experience. I feel that and have always felt that the benefits outweigh the risks. I’m aware that there can be side effects in some cases, but that does not deter me from getting vaccinated*.” Some described the experience of receiving a COVID-19 vaccine as both emotionally and physically traumatic, noting it was “*very apocalyptic, with the guards there and the military there… so that was kind of scary*.”

### HIV, CVD, and COVID-19

#### Influence of HIV on participants’ approaches to COVID-19

Participants were asked whether they approached COVID-19 differently because of having HIV, and to explain their reasoning. Almost two-thirds answered affirmatively. Seventeen did not report any difference in behavior, and four did not provide a response. The proportion of participants who approached COVID-19 differently was higher in Spanish-speaking than English-speaking participants.

##### Themes related to COVID-19 approach being impacted by HIV

The primary theme voiced by participants focused on linkages between living with HV and one’s approach to COVID-19. Many identified a strong desire to get vaccinated for COVID-19 and described taking precautions against possible infection. Many participants explained that they felt at more risk for COVID-19 because they were immunocompromised and described a link between living with HIV and how they approached COVID-19. For example, “*I related that [COVID-19], I connected that to HIV… People may have HIV, [but] they never get tested because they are in denial [...] so they put themselves at risk and they put others at risk. So, I just wanted to make sure that I did not spread anything around because [COVID-19 is] a killer*.” Some participants identified their HIV status as relevant to dealing with COVID-19 because of their self-care skills and the network of healthcare providers and resources accrued as a result of living with HIV. One participant described living with HIV as a “*blessing*” when dealing with infectious disease: “*HIV has really been a blessing because of it and me being susceptible to a lot of other conditions that will result if I did not take care of it, I became extremely [health] conscious*.”

##### Themes related to COVID-19 approach not affected by HIV

Other participants tended to not recognize any connection between living with HIV and their approach to COVID-19. Although many mentioned taking precautions and seeking COVID-19 vaccinations, they did not link it to their HIV status.

#### Impacts of COVID-19 on HIV care

Participants were asked whether the COVID-19 pandemic had affected their HIV-related health care. Almost three-quarters of participants described no change, although 15 participants reported a change in their HIV care due to COVID-19. The proportion of English-speaking participants who did not note a change in their HIV care was higher than among Spanish-speaking participants.

##### Themes among participants perceiving an impact on HIV care

Participants provided four primary reasons for COVID-19 affecting their HIV care, including less access to their healthcare providers due to providers’ increased workloads; pandemic restrictions; and the shift from in-person to virtual healthcare visits. Many participants shared that they suffered from comorbid illnesses such as diabetes and high blood pressure, and that the pandemic amplified the negative effects on their care. One participant shared that they had gone almost a year without visiting their regular clinic. The shift to virtual visits caused pronounced difficulty for many participants, with one describing the process as “*a bit traumatizing for me*,” because the lack of in-person assistance meant they could not get their blood drawn to check their HIV status. Another participant explained: “*In some ways; I had to see my doctors virtually for a long time because they did not have in-person appointments. I’m not too trusting of phone visits, I think there is more trust when it’s face to face*.” Staffing shortages and increased patient loads compromised participants’ ability to find continuous care.

##### Themes among participants perceiving no impact on HIV care

Participants who did not perceive their HIV care as affected by COVID-19 primarily reported a lack of adverse effects or increased benefits on their healthcare. Although some experienced shifts from in-person to virtual care, they did not interpret it negatively. One participant commented that they still had access to in-person HIV care, and that “*the only difference [was] because everybody has the mask up when we are in the clinic, so care-wise, no, just because everybody has to follow a protocol*.” Some participants noted that their immunocompromised status resulted in *increased* benefits, such as being able to receive the COVID-19 vaccine earlier than non-immunocompromised individuals.

#### HIV status and perceptions of the COVID-19 vaccine

Participants described whether and how their HIV status affected COVID-19 vaccine perceptions. One-third of participants believed that their HIV status affected their COVID-19 vaccine perceptions, while the majority did not. A higher proportion of Spanish-speaking, compared to English-speaking, participants did not describe connections between HIV status and COVID-19 vaccine perceptions.

##### Themes related to HIV and the COVID-19 vaccine

Participants primarily described three themes, including trust in the vaccine and a desire to pursue receiving it, fear of vaccine side effects, and prioritizing management of HIV over COVID-19. Many described feeling gratitude for the vaccine and a willingness to seek it. Because of the healthcare networks and resources they had established for their HIV care, they expressed trust in the vaccine and were willing to get vaccinated, even if they did not know its full effects. One participant stated: “*honestly, I did not pay attention to [the impact of the vaccine on HIV medication]. I feel fine, I feel safe. If they give [the vaccine] to me it’s because it’s good*.” Another commented: “*[my HIV status] makes me more accepting of medication for sure and all of those necessary things prescribed by my doctor. I follow my doctor’s orders*.”

Less commonly, participants voiced fear of experiencing excessive side effects associated with their immunocompromised state. They described HIV as their priority and were less concerned about COVID-19, especially if the vaccine posed risks to their health as immunocompromised individuals. Participants noted, “*Even though I do not believe all those things that were said about the vaccines containing foreign elements that were detrimental to the humans, what I do know for sure is that having a compromised immune system because of being HIV positive makes me more susceptible to any foreign agent that may enter my blood or my bloodstream and that is why I do not accept more vaccines*.” Another stated, “*... if a vaccination is going to do something that’s going to mess with [HIV] then I do not want to be vaccinated*.”

Participants who did not agree that their HIV status affected their perceptions about the COVID-19 vaccine generally did not view the two issues as related. A participant stated, “*One thing’s one thing and the other one’s another*.” They tended not to perceive COVID-19 as a serious threat, viewing it differently than HIV, “*I’m just saying it’s normal now and I’m dealing with it. So, it does not affect me any more than the regular part of my– it’s like a regular day now*.”

#### CVD risk and perceptions of the COVID-19 vaccine

Participants were also asked whether and how living with heightened risk of cardiovascular disease (CVD) affected their perceptions about the COVID-19 vaccine. Half of participants answered that CVD did *not* influence their perception of the COVID-19 vaccine, while 12 answered affirmatively. The remaining 18 participants did not provide a response. No Spanish-speaking participants answered in the affirmative, although 14 Spanish-speaking participants did not respond. Among English-speaking participants, less than one-third answered in the affirmative, and over half did not perceive a link between living with CVD risk and perceptions of the COVID-19 vaccine.

##### Themes related to CVD risk and the COVID-19 vaccine

Compared to the influence of one’s HIV status on COVID-19 vaccine perceptions, CVD risk did not seem to be as significant a factor to participants. Almost one-third did not provide any response; those who responded expressed ambivalence due to not identifying as having CVD risk. The remaining participants described three themes, including having multiple medical concerns, elevated COVID-19 risk due to CVD risk, and COVID-19 taking precedence over CVD concerns. Some participants described having too many concurrent health issues to keep track of, and CVD risk was not prioritized. One participant stated that all they could do was make it through the day: “*I just roll with the punches now*.” Participants who identified as being at heightened CVD risk, and who perceived that it affected their COVID-19 vaccine perceptions, expressed a fear of being at higher risk because of their immunocompromised status. Some described measures they were taking to maintain their health, such as: “*I need to be more vigilant about staying healthy, eating correct, and being up to date on the information that’s provided*.” Participants who did not perceive that CVD risk affected COVID-19 vaccination believed COVID-19 took precedence over CVD. For example, one participant stated, “*the COVID vaccine is just something that I feel that as a society, we need to do to protect each other. There was no thought about my condition, as to how COVID would impact my decisions to take tests or vaccinations*.”

#### Sources of COVID-19 vaccine recommendations

COVID-19 vaccinated participants were asked who, if anyone, had recommended vaccination. Participants identified four main sources of influence on their subsequent vaccine decision-making: public health messaging, healthcare providers and resource networks, community networks, and self-volition. Public health messaging included information in the news, public health mandates, government agency websites (such as the CDC), and/or workplace vaccination requirements. According to one participant, a message urging them to get vaccinated and providing instructions “*appeared in my mailbox from the health department*.” Another participant had been court-ordered to receive a COVID-19 vaccine in order to visit their incarcerated grandson. The second category (i.e., healthcare providers and resource networks) included doctors and nurses, support groups for PLWH, and clinical trials. A participant who attended support groups mentioned that “*the doctor would tell the nurse or in groups, there were groups where they said, ‘if you do not have your vaccines, we cannot see you.’*” The third source (i.e., community networks) primarily consisted of friends and family as well as community organizations such as Meals on Wheels. One participant noted that, “*Most of my friends also were advocating to each other that we get vaccinated. We check up on each other. ‘Well, when’s your next shot? Have you gotten it yet?’ We kind of stay on top of it with each other*.” Finally, some participants decided on their own to get vaccinated for COVID-19, and stated they either did not receive outside influence or did not take it into account. As one such participant explained, “*I recommended it... Of course my doctor follows suit, but I usually get there before she does. Am I ready for this next vaccine? Give it to me, please*.”

## Discussion

This qualitative study provides a real-time narrative from PLWH and CVD risks who were extremely vulnerable during the height of the COVID-19 pandemic. Importantly, many were interviewed prior to the widespread availability of COVID-19 vaccines, booster shots, and treatments (e.g., Paxlovid). The significance of this study is further advanced by its contrast to other research studies involving this subpopulation exposed to structural inequities that typically provide secondary data, focusing on the perceptions of community experts rather than the lived experiences of community members (31). The approach of this study provided direct information about the salience of specific social determinants on the health-related behaviors of individuals living with HIV during the pandemic and is consistent with calls for the prioritization of research to better understand the experiences of those exposed to intersecting structural inequities (20). Participants in this study serve as key informants in the identification of patient-centered social and health challenges, providing an opportunity to enhance risk reduction efforts and create novel public health responses to achieve health equity among those disproportionately exposed to SDOH and COVID disparities (9).

### Perceptions of risk

Despite almost all participants reporting that they were fully vaccinated against COVID-19, most voiced continued concerns about their personal risk for COVID-19, primarily due to their understanding of elevated risks due to their immunocompromised health status, the ongoing nature of the pandemic, knowledge that adherence to public health mandates does not eliminate risk, and incurring risks through exposure to unvaccinated individuals or those that are unwilling or unable to adhere to public health recommendations. Those that did not perceive personal risk described feeling protected by COVID-19 vaccines and adhering to public health mandates.

The majority of participants also discussed concerns about community (i.e., geographical community, HIV community) risk; these included the failure of other community members to follow public health mandates, the ongoing nature of the pandemic, community-specific SDOH, and distrust in the medical system and vaccines among community members. Participants in this sample defined community risk differently; many referred to their geographic community, some to their racial/ethnic community, and others focused on their HIV community. Many voiced apprehensions stemming from several SDOH that have received attention in existing literature. These included relying on public transportation (e.g., buses were full of people, inability to socially distance from others), as well as exposure to risks conferred by others (e.g., essential workers, unvaccinated individuals, those that failed to follow public health precautions). Participants also identified several other SDOH that conferred heightened risk, including living in under-resourced neighborhoods, having to leave their community for work, housing issues (e.g., homelessness or living in transitional housing with high resident turnover), and incarceration as contributing to heightened risks for exposure to COVID-19. Other sources of risk reported by participants included perceptions of the ongoing nature of the pandemic, lack of public health information, misinformation, unclear messaging, and varying levels of health literacy and corresponding diligence with respect to following public health recommendations in one’s community that compromise the effectiveness of public health communication as a strategy to mitigate disease risks during public health crises (20). Participants also discussed community risks stemming from distrust in the medical system and hesitancy concerning vaccines in general. Notably, in response to these perceived personal and community risks, many participants reported engaging in proactive counter measures, including adherence to public health mandates as a way of mitigating risk and protecting their health status.

### Sources of COVID-19 information

Themes related to sources of COIVD-19 information centered around the importance of relationships with primary care or other healthcare providers, participation in community groups, support groups and/or community events, and digital access and literacy. These were particularly salient factors with respect to dissemination of COVID-19 public health information as well as vaccine uptake. About two-thirds of the sample identified their primary care or health care provider as among their main sources of COVID-19 information, with many indicating that if their doctor recommended the vaccine, they would receive it. This highlights the importance of leveraging patient-provider relationships and ensuring continuity of care with trusted providers for populations living with chronic illness to achieve public health goals (32). Similarly, community groups, HIV support groups and community events were also frequently reported sources of COVID-19 information. These groups were offered in-person and were only available after safer at home mandates were lifted. Many support groups provided information and invited trusted medical professionals to speak. As such, community and/or support groups created conversations about risks, protective strategies, and provided reliable COVID-19 information. For participants who were hesitant to receive the vaccine because of questions concerning how it might interact with HIV, or who were not aware of the existence of COVID-19 information specifically for PLWH, community groups and events can facilitate the dissemination of reliable, HIV-specific information and address concerns about misinformation that led to confusion about and lack of trust in vaccines and their efficacy. One-third of this sample relied on word of mouth as a main source of COVID-19 public health information. Participants also described relying on the news, primarily television and the radio, for COVID-19 information. Accordingly, one way to capitalize on this common source of information is for providers and community groups to recommend reliable news and social media sources. Understanding where populations exposed to intersecting risks obtain their public health information is critical for mitigating barriers and developing tailored strategies to increase reach and adoption of public health recommendations (20). Results suggest that leveraging these established healthcare networks and resources among populations living with chronic illness may be an important way to reach populations exposed to structural inequities during public health crises.

### Barriers to COVID-19 public health recommendations and vaccine uptake

Most participants faced no difficulties following public health recommendations. Those that did described SDOH-related challenges, including risks incurred due to transportation issues and their living situation (e.g., unsanitary conditions, homelessness). Most participants also reported no problems obtaining COVID-19 tests and vaccines. Barriers reported by those that did experience difficulties included a lack of testing sites in their community, lack of transportation, and concerns about vaccines stemming from their health status (e.g., fears about potential interactions with HIV or HIV medications). Not surprisingly, more Spanish-speaking participants reported difficulty accessing COVID-19 public health information in their preferred language at the onset of the pandemic than English-speaking participants.

Consistent with research noting that digital inequities have the potential to widen existing health disparities (9, 33), participants in this sample also described a lack of internet access and low digital health literacy as barriers to accessing reliable online public health information. Most participants reported having access to the internet; however, only half of participants, mostly English-speaking, used it to obtain COVID-19 public health information. Research has noted that individuals impacted by social determinants who are living with HIV tend to rely on cell phones for internet access, limiting their ability to optimally use telehealth and other health-related digital platforms (33). Those that did have internet access reported using the internet as one of their main sources of public health information. These participants reported accessing reliable sources, including the Los Angeles County Health Department, the Centers for Disease Control, and other official government websites. Those without internet access and/or low digital health literacy would be unlikely to have knowledge of or be able to access online COVID-19 information designed for PLWH, highlighting the importance of mitigating digital inequities as a public health priority (33).

Almost one-quarter of the sample reported having no access to a computer or tablet. Even among those with access to internet-enabled devices, many reported that they experienced difficulties using them. Access to technology and technical literacy are prerequisites for use of digital platforms (33). Lack of digital access and low digital literacy severely restrict access to online public health information as well as participation in health services that utilize digital platforms, threatening continuity of care for PLWH. Although few participants felt that the pandemic had impacted their HIV care, those that did identified the shift from in-person to virtual care as disruptive. Given that misinformation, inconsistent and/or a lack of public health information was reported as a concern among participants, facilitating access to reliable public health information should be a priority in mitigating future public health threats.

About one-third of the sample reported experiencing discrimination during the pandemic; although most did not perceive these experiences as affecting their ability to get COVID-19 information or services, it is unclear to what extent they impacted mental health or other life domains. Participants also reported that past experiences of historical trauma incurred in medical settings and the physical side effects of vaccines influenced their willingness to receive them, suggesting the importance of providing opportunities to discuss these experiences with trusted individuals, obtain information, and ask questions (14). Professionals can address hesitancy by incorporating historical trauma and discrimination in future research and practice (8, 14).

### HIV, CVD, and COVID-19

The primary theme voiced by participants in this sample was that having experience managing a chronic illness like HIV facilitated their vaccine acceptance. About two-thirds of participants said they approached COVID-19 differently because they were living with HIV; they described wanting to receive the vaccine, being health conscious, taking extra precautions, and endorsed testing to minimize the likelihood of spreading the virus to others. About one-third of participants described feeling grateful for and having trust in vaccines because of living with HIV. Compared to the influence of HIV status on COVID-19 vaccine perceptions, participants did not perceive CVD risk to be as significant a factor, in part due to the fact that many participants did not identify themselves as having cardiovascular risk.

This study was subject to limitations. Whereas its strengths include the collection of rich qualitative data, it lacked a mixed-methods design that would also allow for the examination of themes by sociodemographic variables. There is also a lack of generalizability due to the parent study’s sample of convenience and the self-selected sample in the current study. Participants were all receiving care from an HIV provider, limiting the generalizability of results to those without access to care. Similarly, cost has been demonstrated to be a barrier to COVID-19 vaccine uptake in some countries; however, in the U.S., COVID-19 vaccines were available without cost to people 6 months and older, regardless of insurance or immigration status (34–37). Accordingly, barriers to vaccine uptake and care may be underreported by participants in this sample. Participants were also asked if they were “fully vaccinated,” without quantification of the number of boosters, etc., and were subject to self-report, potentially overestimating the number of fully vaccinated individuals.

Notwithstanding these limitations, this study provides initial evidence of the importance of research that identifies SDOH salient to specific vulnerable populations as well as how social determinants intersect (33) by employing methods that allow for the examination of their COVID-19 lived experience. Future implications of this research include the need to identify the impact of traditional SDOH on those living with chronic illness as well as other social determinants that shed light on access to public health information, adherence to public health recommendations, and vaccine uptake among populations exposed to structural inequities to better prepare for future public health threats and eliminate health inequities.

## Data availability statement

The datasets presented in this article are not readily available because this is qualitative data. Requests to access the datasets should be directed to Tloeb@mednet.ucla.edu.

## Ethics statement

The studies involving humans were approved by South General UCLA Institutional Review Board, IRB #21-000762. The studies were conducted in accordance with the local legislation and institutional requirements. The ethics committee/institutional review board waived the requirement of written informed consent for participation from the participants or the participants’ legal guardians/next of kin because verbal informed consent was obtained.

## Author contributions

TL: Conceptualization, Data curation, Formal analysis, Funding acquisition, Investigation, Supervision, Writing – original draft, Writing – review & editing. GA: Data curation, Formal analysis, Project administration, Writing – original draft, Writing – review & editing. EL: Data curation, Formal analysis, Writing – original draft, Writing – review & editing. JM: Data curation, Formal analysis, Writing – original draft, Writing – review & editing. KD: Methodology, Writing – review & editing. MC-S: Data curation, Writing – review & editing. EN-S: Data curation, Writing – review & editing. KR: Data curation, Writing – review & editing. DK: Data curation, Writing – review & editing. AB: Data curation, Writing – review & editing. DN: Writing – review & editing. DC: Writing – review & editing.
